# A Blockchain-Based Location Privacy Protection Incentive Mechanism in Crowd Sensing Networks

**DOI:** 10.3390/s18113894

**Published:** 2018-11-12

**Authors:** Bing Jia, Tao Zhou, Wuyungerile Li, Zhenchang Liu, Jiantao Zhang

**Affiliations:** 1 Inner Mongolia A.R. Key Laboratory of Wireless Networking and Mobile Computing, Hohhot 010021, China; jiabing@imu.edu.cn (B.J.); 31709071@mail.imu.edu.cn (T.Z.); gerile@imu.edu.cn (W.L.); 2 College of Computer Science, Inner Mongolia University, Hohhot 010021, China; 3 Office of Informationization Construction and Management, Nankai University, Tianjin 300071, China; 4 Human Resources Department, Nankai University, Tianjin 300071, China; zjt@nankai.edu.cn

**Keywords:** blockchain, incentive mechanism, crowd sensing network, Internet of Things, privacy protection, cloud computing

## Abstract

Crowd sensing is a perception mode that recruits mobile device users to complete tasks such as data collection and cloud computing. For the cloud computing platform, crowd sensing can not only enable users to collaborate to complete large-scale awareness tasks but also provide users for types, social attributes, and other information for the cloud platform. In order to improve the effectiveness of crowd sensing, many incentive mechanisms have been proposed. Common incentives are monetary reward, entertainment & gamification, social relation, and virtual credit. However, there are rare incentives based on privacy protection basically. In this paper, we proposed a mixed incentive mechanism which combined privacy protection and virtual credit called a blockchain-based location privacy protection incentive mechanism in crowd sensing networks. Its network structure can be divided into three parts which are intelligence crowd sensing networks, confusion mechanism, and blockchain. We conducted the experiments in the campus environment and the results shows that the incentive mechanism proposed in this paper has the efficacious effect in stimulating user participation.

## 1. Introduction

Cloud computing has become a hot technology in research and application in the field of information technology, and it has gradually been applied to people’s daily lives, bringing convenience to people. Crowd sensing networks are becoming common data sensing solutions. Longo Antonella et al. used crowd sensing in the field of teaching [1] and applied crowd sensing for environmental monitoring of several pollutants, such as noise, air, electromagnetic fields, and so on, in an urban area [2]. Alvear Oscar proposed that crowd sensing emerges as a powerful solution to address environmental monitoring, allowing one to control air pollution levels in crowded urban areas [3]. Corradi Antonio et al. used crowd sensing technology in community identification and cooperative task execution [4]. Habibzadeh Hadi et al. conducted a thorough study of both types of sensors and drew conclusions about which one becomes a favorable option based on a given application platform [5]. Panichpapiboon Sooksan et al. explored the possibility of using only the built-in sensors of off-the-shelf smartphones for traffic density estimation [6]. Cortellazzi Jacopo et al. presented an extension of the general-purpose ParticipAct platform, an MCS application developed by the University of Bologna, focused on the needs of people with impaired mobility. The goal is to specialize ParticipAct to enable a crowd sensing platform that guarantees a solid support for their lifetime allowing for reviewing and sharing opinions regarding public and private places and architectonic barriers of a city area [7].

Crowd sensing not only enables users to collaborate on large-scale awareness tasks but also provides users with the type of cloud platform, social attributes, and other information. Like personal relationships, the level of trust determines how we control privacy in the environment of IoT. As IoT devices become more connected, more data will be shared between people, companies, governments, and ecosystems. Sensors, devices, data, computers, and cloud connections rely heavily on established trust relationships. Connecting more types of IoT devices (equivalent to adding intrusion points) will increase the threat of established systems, increasing overall security risks. If there is no ability to limit privacy settings [8,9], it is difficult to establish a trust relationship with an IoT system or device. Many manufacturers want to accelerate the adoption of IoT products, but security is often the challenge they face. Many IoT devices will continue to face security risks without a security mechanism. Nowadays, mobile devices such as mobile phones and tablet computers have become part of life. This provides the conditions for the development of crowd sensing networks. Traditional sensors have high prices and are not portable. Crowd sensing networks use sensors carried by mobile phones to collect sensing data and can effectively save costs. However, the most important issue with the use of mobile phones to collect sensing data is privacy leakage. User engagement and correctness of information are two of the most important issues in crowd sensing networks. The problems caused by privacy leakage lead to low user involvement, or users intentionally upload false information in order to protect their privacy information. Most of the incentive mechanisms are based on user data quality to motivate users to participate. The higher the quality of data provided by users, the more rewards the server will return to users. Wen et al. proposed “Quality-Driven Auction-Based Incentive Mechanism for Mobile Crowd Sensing” [10], and Guo et al. proposed “TaskMe: Toward a Dynamic and Quality-Enhanced Incentive Mechanism for Mobile Crowd Sensing” [11] and “TaskMe: a cross-community, quality-enhanced incentive mechanism for mobile crowd sensing” [12]. In addition, some scholars have proposed other incentive mechanisms. Zhang et al. proposed an incentive mechanism with a moral hazard. They adopted the performance-related contract to incentivize users to turn on their sensors and allow data collecting for the principle [13]. Jiajun Sun proposed a behavior-based incentive mechanism for crowd sensing applications with budget constraints by applying sequential all-pay auctions in mobile social networks (MSNs) [14]. These incentives have certain effects, but the authors ignore the most fundamental issue: privacy protection. The blockchain [15,16] is a chained data structure that was designed by Nakamoto Sutoshi in 2008. It combines data blocks in a sequential manner in chronological order and ensures that information is not forged and modified through encryption technology. It has the characteristics of decentralization, openness, autonomy, information modification, and anonymity. Therefore, various industries have gradually applied blockchain technology to protect users’ information security.

We considered the issue of privacy protection and designed a blockchain-based incentive mechanism. We used the blockchain protection mechanism to protect the user’s privacy information, giving certain incentives to motivate users to participate in sensing tasks. The rest of this paper is organized as follows. Section 2 introduces the blockchain-based incentive framework in crowd sensing networks. Section 3 describes the confusion mechanism and confusion mechanism algorithm (CMA). Section 4 reports the structure of the Merkle tree and motivation strategy. Finally, we conclude our work in Section 5 and Section 6.

## 2. Blockchain-Based Incentive Framework in Crowd Sensing Networks

The extension of blockchain technology was born in Bitcoin. It is not just for Bitcoin. Since blockchain technology has unique timestamps, a chain structure, asymmetric encryption, etc., the data stored in the blockchain has the characteristics of being unforgeable and falsified. This non-tamperable characteristic has great significance in the fields of smart cities, intelligent transportation, Internet of Things, finance, finance, trade, trade, credit, etc. It marks the beginning of a truly trustworthy Internet [17].

**Figure 1:**
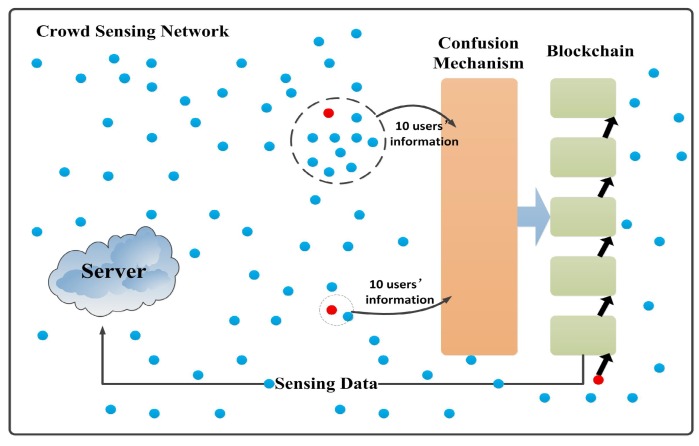
A blockchain-based incentive framework in a crowd sensing network.

As shown in Figure 1, the network structure of blockchains [18] can be divided into three parts: intelligence crowd sensing networks, confusion mechanisms, and blockchain. There are two types of nodes in crowd sensing networks. One is an ordinary user node carrying user information, and the other is a miner node. The miner node does not carry any information. Its main role is to mine the new block space. The main function of a server in a crowd sensing network is to publish task information and receive sensing data from the blockchain. The role of the confusion mechanism is to process the sensing data of the nodes in the crowd sensing network to achieve the effect of obfuscating the node data and to prevent the leakage of the node’s privacy information. Blockchain includes characteristics of decentralization and transparent information. Using a blockchain structure can prevent user information from being tampered with and protect user information privacy. It can strengthen the privacy protection of information. After introducing the function of each parts, we describe the workflow of the framework. First, the server issues a sensing task. The nodes in the crowd sensing network accept the task. The node collects the sensing data and enters the confusion mechanism for information protection. Secondly, there are a certain number of miners which are red nodes, in Figure 1, responsible for opening up new block spaces. The confusion mechanism creates a group that includes 10 users and a miner. The group is used to handle the data of these users. After user data is stored in the blockchain, the blockchain will give the user the virtual coin as a reward. The more frequency with which the user participates in the task, the more reward coins there will be. The virtual coin can be exchanged for cash. Finally, the blockchain stores the information handled by the confusion mechanism, strengthens the protection on it, and sends the sensing data to the server.

## 3. Confusion Mechanism

### 3.1. Information Coding Definition

There are many algorithms for handling user privacy protection. The classical privacy protection algorithm is the K-anonymity algorithm [19]. These algorithms have one commonality: fuzzing user information. When the server is attacked by a malicious attacker, the attacker can find many users that meet the conditions, such that the attacker cannot distinguish the target user and ensure the security of the user. In this paper, we use coded methods to protect user information [20]. Each part of the user information is encoded, and the obtained coding information is combined using a plurality of coding methods to form a final user code. Since the privacy of the location information is mainly considered in this paper, the ID of the user and the perceptual information of the user are not encoded. We will then introduce the coding method of each part [21,22,23].

1. Longitude coding method: Change the longitude to a 9-digit number (remove the extra part after the decimal point) and change the number to a vector $\vec{Lo}$. The calculation is as shown in Equations (1) and (2). Converting $\vec{Lo^{\prime }}$ into a number is the encoding of longitude. Similarly, as shown in Equation (3), when decoding $\vec{Lo^{\prime }}$, simply multiply by $Q^{-1}$ to obtain the original vector $\vec{Lo}$ [24,25].
  (1) $$Q=\left[\begin{array}{cccc} Q_{1,1} & Q_{1,2} & \cdots & Q_{1,9}\\ Q_{2,1} & Q_{2,2} & \cdots & Q_{2,9}\\ \vdots & \vdots & \ddots & \vdots \\ Q_{9,1} & Q_{9,2} & \cdots & Q_{9,9} \end{array}\right]$$
  (2) $$\vec{Lo}\times Q=\vec{Lo^{\prime }}$$
  (3) $$\vec{Lo^{\prime }}\times Q^{-1}=\vec{Lo}.$$
2. Latitude coding method: Latitude code and longitude code are the same. Change the latitude to an 8-digit number (remove the extra part after the decimal point) and change the number to a vector $\vec{La}$. The calculation is as shown in Equations (4) and (5). Converting $\vec{La^{\prime }}$ into a number is the encoding of longitude. Similarly, as shown in Equation (6), when decoding $\vec{La^{\prime }}$, simply multiply by $P^{-1}$ to obtain the original vector $\vec{La}$.
  (4) $$P=\left[\begin{array}{cccc} P_{1,1} & P_{1,2} & \cdots & P_{1,8}\\ P_{2,1} & P_{2,2} & \cdots & P_{2,8}\\ \vdots & \vdots & \ddots & \vdots \\ P_{8,1} & P_{8,2} & \cdots & P_{8,8} \end{array}\right]$$
  (5) $$\vec{La}\times P=\vec{La^{\prime }}$$
  (6) $$\vec{La^{\prime }}\times P^{-1}=\vec{La}.$$
3. Age information coding: The age information is encoded as shown in Equation (7). $Key_{a}=\left(\alpha ,\beta \right)$ and $\hat{age}$ is the age code. The decoding method is as shown in Equation (8) [26].
  (7) $$\hat{age}=age\times \alpha +\beta$$
  (8) $$age=(\hat{age}-\beta )÷\alpha .$$
4. Gender information coding: Gender information is relatively simple. We directly define its encoding. Male is encoded as 01 and female is encoded as 02.
5. Hobby information coding: As shown in Equation (9), we use an affine cipher algorithm [27,28] to encode hobby messages in Equation (9). The key is $Key_{h}=\left(\mu ,\nu \right)$. fun(hobby) is used to convert an alphabet letter to a corresponding number. Its decoding is shown as Equation (10).
  (9) $$\hat{f}\left(hobby\right)=\left[fun\left(hobby\right)\times \mu +\nu \right]mod\left(26\right)$$
  (10) $$fun\left(hobby\right)=\lambda \times (\hat{f}\left(hobby\right)-\nu )mod\left(26\right)$$
  $$\lambda =\mu^{-1}mod\left(26\right).$$
6. Occupation information coding: Occupation information coding is the same with hobby information coding in Equation (11). The key is $Key_{o}=\left(\mu^{{}^{\prime }},\nu^{{}^{\prime }}\right)$. fun(occupation) is used to convert an alphabet letter to a corresponding number. Its decoding is shown as Equation (12).
  (11) $$\hat{f}\left(occupation\right)=\left[fun\left(occupation\right)\times \mu^{{}^{\prime }}+\nu^{{}^{\prime }}\right]mod\left(26\right)$$
  (12) $$fun\left(occupation\right)=\gamma \times (\hat{f}\left(occupation\right)-\nu^{{}^{\prime }})mod\left(26\right)$$
  $$\gamma =\mu^{{}^{\prime }-1}mod\left(26\right).$$

### 3.2. Coding And Decoding Algorithm

**Figure 2:**
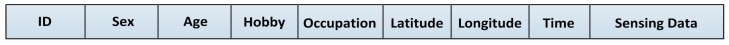
The order of the user information.

Based on the above, we summarize two algorithms: the Confusion Mechanism Encode Algorithm (CMA-E) and the Confusion Mechanism Decode Algorithm (CMA-D). The order of the user information is as shown in Figure 2. Algorithm 1 is an encoding algorithm that encodes the information obtained by the user, and the coding of each part is spliced to form a code carrying all the user information. Algorithm 2 is a decoding algorithm that decodes the encoded information formed by the user and restores the user information [29].

**Algorithm 1** Confusion Mechanism Encode Algorithm (CMA-E)**Require:** User information **Ensure:** Encoded user information  $\vec{Lo}\leftarrow$ longitude;  $\vec{La}\leftarrow$ latitude;  $Key_{a}=\left(\alpha ,\beta \right)$;  $Key_{h}=\left(\mu ,\nu \right)$;  $Key_{o}=\left(\mu^{{}^{\prime }},\nu^{{}^{\prime }}\right)$;  Matrix P and matrix Q;  **if** sex=man **then**   $sex^{{}^{\prime }}$←01;  **else**   $sex^{{}^{\prime }}$←02;  **end if**  $\vec{Lo^{\prime }}\leftarrow \vec{Lo}\times Q$;  $\vec{La^{\prime }}\leftarrow \vec{La}\times P$;  $\hat{age}\leftarrow age\times \alpha +\beta$;  $fun\left(hobby\right)\leftarrow \lambda \times (\hat{f}\left(hobby\right)-\nu )mod\left(26\right)$;  $fun\left(occupation\right)=\gamma \times (\hat{f}\left(occupation\right)-\nu^{{}^{\prime }})mod\left(26\right)$;  Combine coding information in order  which as $ID,sex^{{}^{\prime }},\hat{age},\hat{f}\left(hobby\right),\hat{f}\left(occupation\right),Lo^{{}^{\prime }},La^{{}^{\prime }},sensingdata,Time$

**Algorithm 2** Confusion Mechanism Decode Algorithm (CMA-D)**Require:** Encoded user information **Ensure:** User information  Split coding information in order, which as $ID,sex^{{}^{\prime }},$  $\hat{age},\hat{f}\left(hobby\right),\hat{f}\left(occupation\right),Lo^{{}^{\prime }},La^{{}^{\prime }},sensingdata,Time$  $Key_{a}=\left(\alpha ,\beta \right)$;  $Key_{h}=\left(\mu ,\nu \right)$;  $Key_{o}=\left(\mu^{{}^{\prime }},\nu^{{}^{\prime }}\right)$;  Matrix $P^{-1}$ and matrix $Q^{-1}$;  **if**
$sex^{{}^{\prime }}$ = 01 **then**   sex←man;  **else**   Data does not exist;  **end if**  **if**
$sex^{{}^{\prime }}$ = 02 **then**   sex←woman;  **else**   Data does not exist;  **end if**
  $\vec{Lo}=\vec{Lo^{\prime }}\times Q^{-1}$, $\vec{La}=\vec{La^{\prime }}\times P^{-1}$;  $age\leftarrow (\hat{age}-\beta )÷\alpha$;  $fun\left(hobby\right)=\lambda \times (\hat{f}\left(hobby\right)-\nu )mod\left(26\right)$;  $fun\left(occupation\right)=\gamma \times (\hat{f}\left(occupation\right)-\nu^{{}^{\prime }})mod\left(26\right)$;

## 4. The Application of Blockchain in Crowd Sensing Networks

Blockchain has the following characteristics:

1. Decentralization: Due to the use of distributed accounting and storage, there is no centralized hardware or management organization. The rights and obligations of any node are equal.
2. Openness: The system is open. In addition to the private information of the parties to the transaction being encrypted, the data of the blockchain is open to everyone. Anyone can query the blockchain data and develop related applications through the open interface. The entire system information is highly transparent.
3. Autonomy: Blockchain adopts consensus-based specifications and protocols (such as a set of transparent and transparent algorithms) to enable all nodes in the entire system to exchange data freely and securely in a trusted environment, so that the trust of "people" can be changed. Become a trust in the machine, and any human intervention does not work.
4. Information cannot be tampered with: Once the information is verified and added to the blockchain, it is stored permanently. Unless more than 51% of the nodes in the system can be controlled at the same time, the modification of the database on a single node is invalid, so the data stability and reliability of the chain is extremely high.
5. Anonymity: Since the exchange between nodes follows a fixed algorithm, the data interaction does not need to be trusted (the program rules in the blockchain will judge whether the activity is valid), so the counterparty does not need to open the identity to let the other party generate itself. Trust is very helpful for the accumulation of credit.

**Figure 3:**
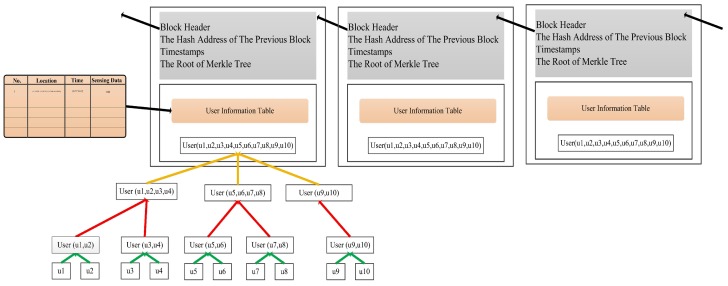
The application of blockchain in a crowd sensing network.

In this paper, we have changed the structure of the blockchain. It is shown as Figure 3. After the confusion mechanism handles the information. The information of users was put to the blockchain structure for storage. The blockchain is linked by a hash value. Each block is composed of a block header and a block body. The block header includes the hash value of the previous block, the timestamp, and the root hash value of the Merkle tree. The block body consists of a Merkle tree and a table which stores user location and sensing data. The reasons for using the blockchain structure after the confusion mechanism are as follows: Firstly, the main function of the confusion mechanism is to encode the user’s information, but it cannot guarantee that the user’s information is not falsified. The blockchain has the function of tamper-proof, so the blockchain technology is an effective method to protect a user’s information. Secondly, blockchain is a distributed database. We use blockchain to store data information because it can retain the characteristics of the blockchain itself, such as decentralization, openness, self-control, information that cannot be tampered with, and anonymity.

### 4.1. The Structure of the Merkle Tree and Motivation Strategy

The Merkle tree [30,31] is built from the bottom up. We hash the user information and store the hash in the corresponding leaf node shown in Figure 3. In this paper, we use the double-SHA256 encryption hash algorithm to hash the user information. The formula is as follows:

(13) $$H∼u_{i}=SHA256(SHA256\left(Information_{ui}\right).$$

By concatenating the hash values of adjacent leaf nodes and hash it, the two leaf nodes are concatenated as a parent node, and that is repeated until one node remains at the top, which is the root of the Merkle tree. Thus, through the Merkle tree, all of the information of the nodes in the group is concatenated into a DataGroup, which is verified by blockchain. If the verification is successful, it will generate the block into the blockchain. Each block has 10 virtual currencies. After the verification is successful, the block distributes the 10 virtual currencies equally to 10 users in order to motivate them to participate more in the task [32,33]. The above description can be summarized as Algorithm 3.

**Algorithm 3** Building the Merkle Tree and Currency Allocation**Require:** 9 encoded user information and a minner ($u_{1},u_{2}...u_{9}$ and miner) **Ensure:** Merkle tree and currency allocation  **repeat**   Continue to find new block; **until** miner find the new block $u_{10}\leftarrow miner$ **for**
i=1;i<=10;i++
**do**   $H\left(u_{i}\right)\leftarrow SHA256(SHA256\left(Information_{ui}\right)$  **end for**
  **for**
i=1;i<=9;i+2
**do**   $H\left(u_{i},u_{i+1}\right)\leftarrow SHA256(H\left(u_{i}\right),H\left(u_{i+1)}\right)$  **end for**
  **for**
i=1;i<=9;i+4
**do**   **if**
i<=8
**then**    $H\left(u_{i},u_{i+1},u_{i+2},u_{i+3}\right)\leftarrow SHA256\left(H\left(u_{i},u_{i+1}\right),H\left(u_{i+2},u_{i+3}\right)\right)$   **else**    $H\left(u_{i},u_{i+1}\right)\leftarrow SHA256(SHA256(H\left(u_{i}\right),H\left(u_{i+1))}\right)$   **end if**  **end for**  $H\left(u_{1},u_{2},u_{3},u_{4},u_{5},u_{6},u_{7},u_{8},u_{9},u_{10}\right)\leftarrow SHA256\left(H\left(u_{1},u_{2},u_{3},u_{4}\right),H\left(u_{5},u_{6},u_{7},u_{8}\right),H\left(u_{9},u_{10}\right)\right)$  **if**
$H(u_{1},u_{2},u_{3},u_{4},u_{5},u_{6},u_{7},u_{8},u_{9},u_{10})$ is legal **then**   Reward←10   **for**
j=1;j<=10;j++
**do**    $Reward_{u_{j}}\leftarrow 1$   **end for**  **else**   Reassemble data;  **end if**

### 4.2. User Information Table

**Figure 4:**
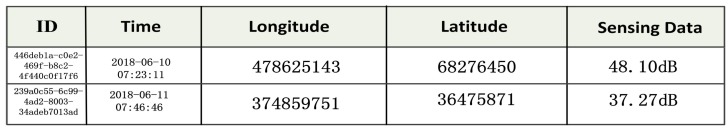
User information table.

The main function of the user information table is to send the user’s perception information to the server to complete the sensing task [34]. As shown in Figure 4, in the user information table, we only store the user’s number, location information, sensing data, and acquisition time. In order to ensure the security of the user’s information, the user’s location information and the user’s acquisition time are the information processed by the confusion mechanism. When the block meets the verification of the blockchain, the blockchain sends this table to the server. Because the user information recorded in the blockchain is not modifiable, when the server finds that there is a problem with the user, the validity of the user information can be verified through the blockchain, thereby removing malicious users.

## 5. Experiment and Result

### 5.1. Set up

The task of this experiment is to collect noise information in the environment. The experiment employed Android Studio and Eclipse as the experimental environment. We use Android Studio [35] to write the app because it is convenient for users to carry and collect environmental information. The environmental information collected in this experiment mainly includes collection time, GPS information, noise information, and basic user information. The collection method is divided into two kinds for comparison. One is the traditional mode which collects and transmits the data directly to the server without encryption protection and the other is the proposed mode, where the data is encrypted by the CMA algorithm and stored. Parameters are shown in Table 1. Encrypted data forming blocks were added to the blockchain to prevent tampering.

This experiment used Eclipse to write the server responsible for processing the collection data (the construction of data blocks, user data storage, etc.). The participants participated in the acquisition of noise information in the environment using their Android mobile phone, and the collected data was uploaded to the server. We conducted a total of 10 acquisitions. Each time, the user voluntarily selected the acquisition mode (proposed or traditional mode), collecting only one piece of data per person at a time.

### 5.2. Result and Analysis

**Figure 5:**
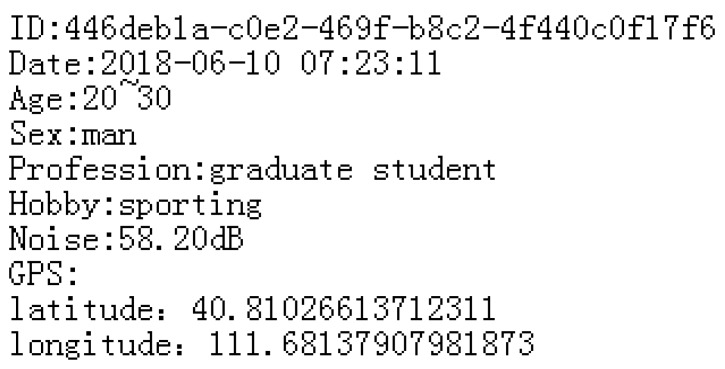
The data collected by the traditional mode.

**Figure 6:**
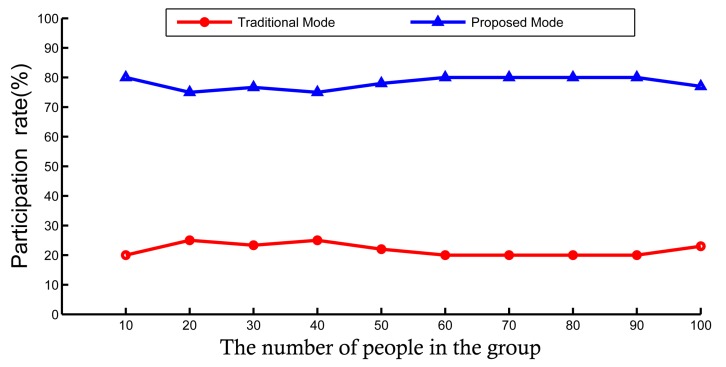
Participation rate.

**Figure 7:**
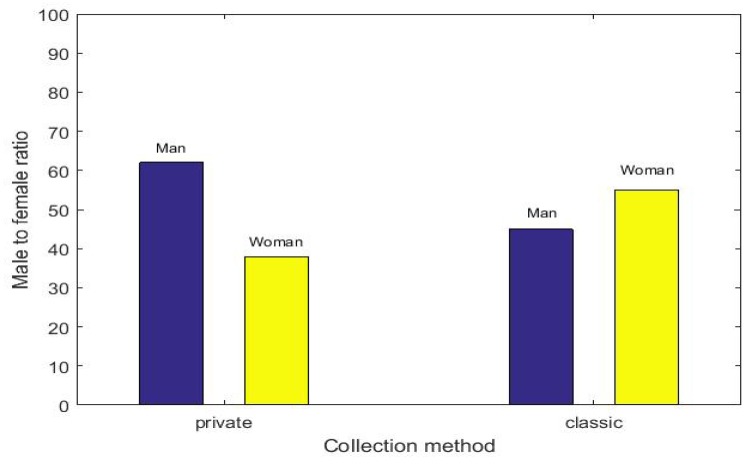
Male-to-female ratio.

In the experiment, we collected a total of 100 pieces of data. The data that we collected is shown in Figure 5. The results are shown in Figure 6. The x-axis indicates that we randomly selected 10, 20, 30, 40, 50, 60, 70, 80, 90, and 100 from all data. The participation rate represents the ratio in each set. There are obviously more people who chose the proposed than those who chose the traditional mode. People who chose the proposed are stableat 80%. The male-to-female ratio of the two methods is shown in Figure 7. Women are the majority in the traditional method, and males account for the majority in the proposed method. This shows that men are more concerned about the importance of privacy protection. Experimental results show that the method proposed in this paper has a certain incentive effect. Algorithm 3, building the Merkle tree and currency allocation, and the Algorithm 3 has three parallel loops, so its time complexity is Tn=O(3n). The time complexity of the blockchain is Tn=O(1). Therefore, the time complexity of the proposed mode is O(n), which is less time-complex compared with the other method.

## 6. Conclusions

In this paper, we propose a mixed incentive mechanism that considers privacy protection and virtual credit. The data processed by the confusion mechanism can be prevented from being attacked. In addition, the addition of blockchain ensures that data is not tampered with by others. Compared with the traditional model, the proposed model in this paper can significantly stimulate user participation. However, due to the small scope of the experiment and the small number of samples, the experimental results obtained may be one-sided. Therefore, our future work will expand the scope of the experiments and the experimental data for a more complete judgment. We will also continue to improve the experimental algorithm and achieve the best protection effect.

## Figures and Tables

**Table 1:** The simulation parameters.

No.	Parameter	Value
1	$Key_{a}=\left(\alpha ,\beta \right)$	$Key_{a}=\left(2,3\right)$
2	$Key_{h}=\left(\mu ,\nu \right)$	$Key_{h}=\left(3,5\right)$
3	$Key_{o}=\left(\mu^{{}^{\prime }},\nu^{{}^{\prime }}\right)$	$Key_{o}=\left(6,2\right)$
4	*Q*	$Q=\left[\begin{array}{cccc} 1 & 0 & \cdots & 0\\ 0 & 2 & \cdots & 0\\ \vdots & \vdots & \ddots & \vdots \\ 0 & 0 & \cdots & 9 \end{array}\right]$
5	*P*	$P=\left[\begin{array}{cccc} 1 & 0 & \cdots & 0\\ 0 & 3 & \cdots & 0\\ \vdots & \vdots & \ddots & \vdots \\ 0 & 0 & \cdots & 2n-1 \end{array}\right]$
6	$Q^{-1}$	$Q=\left[\begin{array}{cccc} 1 & 0 & \cdots & 0\\ 0 & 1/2 & \cdots & 0\\ \vdots & \vdots & \ddots & \vdots \\ 0 & 0 & \cdots & 1/9 \end{array}\right]$
7	$P^{-1}$	$P=\left[\begin{array}{cccc} 1 & 0 & \cdots & 0\\ 0 & 1/3 & \cdots & 0\\ \vdots & \vdots & \ddots & \vdots \\ 0 & 0 & \cdots & 1/(2n-1) \end{array}\right]$
8	α	2
9	β	5
